# Etiologies of intermediate uveitis in a tertiary center: an age-oriented medical assessment?

**DOI:** 10.1186/s12348-025-00563-w

**Published:** 2025-12-13

**Authors:** P. Mansuy, T. El-Jammal, R. Jacquot, T. Mathis, L. Kodjikian, Pascal Sève

**Affiliations:** 1 Université Claude Bernard Lyon 1, Lyon, France; 2 Department of Ophthalmology, Hôpital de la Croix-Rousse, Hospices Civils de Lyon, Université Claude Bernard Lyon 1, Lyon, France; 3 Department of Internal Medicine, Hôpital de la Croix-Rousse, Hospices Civils de Lyon, Université Claude Bernard Lyon 1, 103 Grande Rue de la Croix-Rousse, Lyon, 69004 France; 4 Laboratory of Tissue Biology and Therapeutic Engineering, CNRS UMR5305, Claude Bernard Université Lyon 1, Lyon, France; 5 MATEIS Laboratory, UMR CNRS 5510, INSA, Université Lyon 1, Villeurbanne, France; 6 Research on Healthcare Performance (RESHAPE), INSERM U1290, Université Claude Bernard Lyon 1, Lyon, France

**Keywords:** Intermediate uveitis, Pars planitis, Sarcoidosis, Multiple sclerosis, Lymphoma

## Abstract

**Purpose:**

To analyze the distribution of intermediate uveitis (IU) etiologies by age in order to guide the diagnostic approach.

**Methods:**

We conducted a monocentric retrospective study of 126 adult patients referred to an internal medicine department at a tertiary center with a diagnosis of intermediate uveitis of unknown cause between July 2002 and April 2023.

**Results:**

Among the 126 patients, most were women (*n* = 77; 61%), with a median age of 49 [33;62] years; 113 (90%) were Caucasian. Idiopathic IU was the most common etiology across all age groups. In patients under 30 years old (*n* = 20), idiopathic IU was observed in 10 cases (50%), followed by pars planitis in 7 cases (35%) and multiple sclerosis in 2 cases (10%). In the 30–60-year group (*n* = 71), idiopathic IU remained the most frequent (*n* = 34, 48%), followed by sarcoidosis (*n* = 15, 21%) and lymphoma (*n* = 5, 7%). Other etiologies included pars planitis (*n* = 3), multiple sclerosis (*n* = 3), and infections (*n* = 2). After 60 years (*n* = 35), idiopathic IU was again the most prevalent (*n* = 22, 63%), followed by lymphoma (*n* = 6, 17%) and sarcoidosis (*n* = 5, 14%). Infectious diseases were observed in 2 cases.

**Conclusions:**

The etiologic distribution of IU varies with age. Before 30, idiopathic IU, pars planitis, and multiple sclerosis are the most common etiologies. After 30, idiopathic IU, sarcoidosis, and lymphoma become more prevalent, with lymphoma appearing after 45. After 60, lymphoma is more common than sarcoidosis. These findings support the use of an age-stratified diagnostic approach in the evaluation of IU.

## Introduction

Intermediate uveitis (IU) was defined in 2005 by the Standardization of Uveitis Nomenclature (SUN) as a subgroup of uveitis in which inflammation is primarily localized in the vitreous and peripheral retina [1]. Although relatively uncommon as an isolated entity, IU accounts for approximately 0.9 to 22.9% of all uveitis cases, depending on the population and referral patterns [2]. It preferentially affects young adults, most often between the second and fourth decades, and frequently presents as bilateral disease [2].

Idiopathic IU represents the most frequent etiology, accounting for 53% to 75.5% of cases across various studies [2]. However, IU may also be associated with systemic and infectious diseases. In Western countries, inflammatory conditions such as sarcoidosis and multiple sclerosis (MS) are among the most common identifiable causes, followed by infectious diseases [3–6]. Infections are more common in developing countries and may be bacterial (e.g., tuberculosis, syphilis, Lyme disease), parasitic (toxocariasis, toxoplasmosis), or viral. In patients older than 40 years, intra-ocular lymphoma must always be suspected, as it often masquerades as chronic IU [7]. Indeed, a cluster of conditions known collectively as masquerade syndromes may imitate IU, including malignant entities such as primary vitreoretinal lymphoma, uveal melanoma and ocular metastases [8]. Pars planitis is a distinct subset of idiopathic IU, characterized by the presence of snowballs and/or snowbanking, without any sign of systemic disease [9]. It mainly affects children and adolescents, typically presents as bilateral, chronic and accounts for up to 26.7% of pediatric uveitis cases [10]. Compared to adults, children tend to have a more severe disease course and a poorer visual prognosis, requiring early and aggressive treatment to prevent vision loss [10].

As with other anatomical types of uveitis, the diagnostic approach requires consideration of demographic data, medical history, physical examination, and targeted laboratory and imaging testing [11]. Because some etiologies reflect serious systemic disease, the diagnostic work-up must be prompt and appropriately focused and recognizing age-related trends could further streamline investigations and lead to a timely diagnosis.

In this study, we aimed to analyze the distribution of IU etiologies according to age in a cohort of IU patients referred to a tertiary center for etiological evaluation. Our objective was to refine the diagnostic approach by identifying age-specific patterns of IU etiologies.

## Patients and methods

### Study design and population

**Fig. 1:**
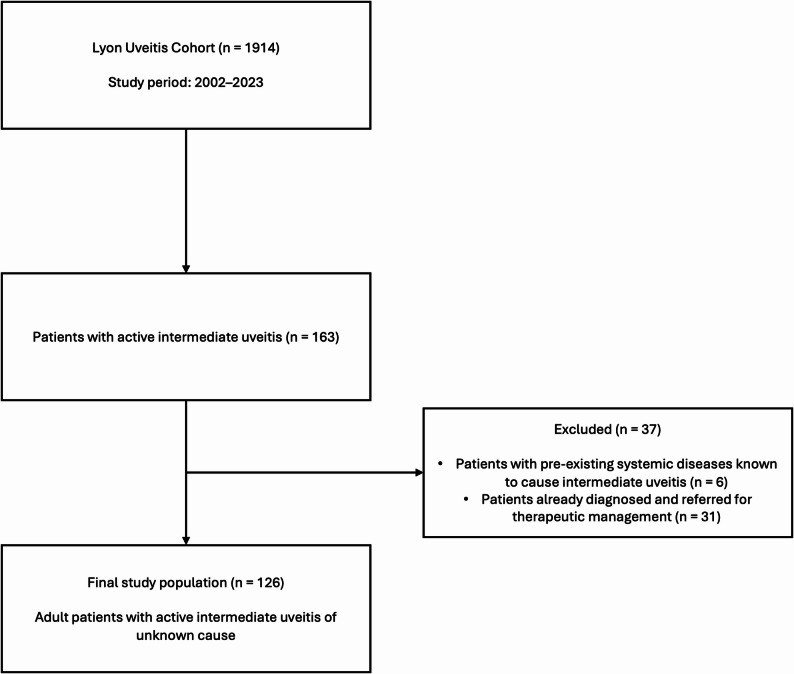
Flowchart of patient selection

We analyzed data from the Lyon Uveitis cohort, focusing on adult patients referred for IU to the Internal Medicine Department of Croix-Rousse Hospital between July 2002 and April 2023. All patients were referred by an ophthalmologist - either from hospital settings or private practice - when no etiological diagnosis of uveitis could be identified. Only patients with active intraocular inflammation at the time of referral were included. Patients with pre-existing systemic diseases known to cause uveitis and patients already diagnosed by ophthalmologist and referred for therapeutic management were excluded. Patients with missing data were also excluded from analyses. Figure 1 presents the flowchart of patient selection and exclusion for the study.

### Diagnostic work-up

All patients underwent a standard screening protocol for uveitis, which included [12]:

- A tuberculin skin test or an interferon-gamma release assay (IGRA)
- C-reactive protein (CRP) and erythrocyte sedimentation rate (ESR)
- A complete blood count (CBC)
- Serological tests for syphilis
- Serum angiotensin-converting enzyme (ACE) levels
- A chest X-ray and/or a chest computed tomography (CT) scan.

Additional examinations were performed at the clinician’s discretion, in accordance with the recommendations of the ULISSE study [12]. The diagnostic screening for sarcoidosis included conjunctival or skin biopsies, if there were clinically suggestive features. Some patients underwent a minor salivary gland biopsy (MSGB), a transbronchial lung biopsy, a bronchoalveolar lavage (BAL), brain magnetic resonance imaging (MRI), or nuclear imaging with 18 F-fluorodeoxyglucose positron-emission tomography/computed tomography (18 F-FDG PET/CT), a whole-body imaging modality that detects hypermetabolic foci, enabling visualization of inflammatory, granulomatous, and neoplastic lesions. This work-up was completed in some patients by an anterior chamber paracentesis (ACP) with interleukin-6 and interleukin-10 measurements, a vitreous biopsy, and/or a lumbar puncture, when appropriate.

### Clinical and demographic data collection

The medical records of each patient were reviewed retrospectively to collect demographic information (sex, ethnicity, age at diagnosis), follow-up duration, uveitis characteristics (anatomic type, laterality, chronicity), specific ophthalmological features (anterior segment involvement, snowballs, snowbanks, vasculitis, papillitis, and macular edema, confirmed, if necessary, by optical coherence tomography [OCT] and/or fluorescein angiography [FA]). Additional data included the positivity or negativity of additional investigations performed (chest X-ray, chest CT scan, MRI of the brain, 18 F-FDG PET/CT, anterior chamber paracentesis with IL-6/IL-10 measurement, vitreous biopsy). A result was deemed positive according to modality-specific criteria. For chest X-rays, the Scadding classification was applied [13]. Chest CT scans were considered normal or consistent with sarcoidosis when they showed bilateral, non-compressive, non-necrotic hilar or mediastinal lymphadenopathies >1 cm, perilymphatic nodules, or other parenchymal abnormalities typical of pulmonary sarcoidosis [14]. Beyond chest imaging, results were considered positive when brain MRI revealed demyelinating plaques compatible with multiple sclerosis or lesions suggestive of lymphoma; when ¹⁸F-FDG PET/CT showed focal hypermetabolic uptake compatible with sarcoidosis; when the aqueous humour demonstrated an IL-10/IL-6 ratio >1; or when malignant cells were identified on vitreous cytology.

The diagnosis of intermediate uveitis followed the criteria defined by the Standardization of Uveitis Nomenclature (SUN), and uveitis was considered chronic if a rapid relapse (within less than 3 months) occurred after discontinuation of treatment, in accordance with SUN criteria [1].

### Diagnostic criteria for etiologies

For etiological diagnosis, we used Abad’s modified criteria for sarcoidosis-related IU [15], the revised 2017 McDonald criteria for multiple sclerosis-associated uveitis [16], and the SUN criteria for the diagnosis of pars planitis [9]. For Lyme disease-associated IU, diagnosis was based on a positive ELISA and Immunoblot serology, exclusion of differential diagnoses, and the effectiveness of a trial course of antibiotic therapy [17]. Tuberculosis-related IU was diagnosed using the Collaborative Ocular Tuberculosis Study (COTS) criteria [18]. The diagnosis of oculocerebral and primary intraocular lymphoma was established through cytopathological analysis of vitreous samples obtained by diagnostic pars plana vitrectomy.

### Statistical analysis

Data analysis was conducted using Microsoft Excel. Patients were stratified into three age groups (< 30, 30–60, ≥ 60 years); for each group we described the etiologic distribution, sex ratio, ethnicity, uveitis features and associated clinical signs. Descriptive cross-tabulations were used to assess the potential diagnostic contribution of brain MRI in the absence of neurological symptoms, and of chest CT when chest X-rays were normal. Differences in etiologic distribution among age groups were assessed using the Chi-square test. Linear trend analyses (Pearson correlation) were performed to evaluate age-related variation for lymphoma and pars planitis. The frequency of sarcoidosis between middle-aged patients and the combined younger and older groups was compared using Fisher’s exact test.

### Ethics statement

The study received approval from the local ethics committee in February 2019 (No. 19–31) and was registered on clinicaltrials.gov (NCT03877575).

## Results

### Patients characteristics

Among the 1,914 patients included in the cohort, 163 were adults with intermediate uveitis. Six of them had a pre-existing systemic condition known to be a potential cause for intermediate uveitis [multiple sclerosis (*n* = 3), sarcoidosis (*n* = 2), and inflammatory bowel disease (*n* = 1)]. Additionally, 31 patients had already been diagnosed by their ophthalmologist and were referred for therapeutic [Fuchs’ heterochromic cyclitis (*n* = 4), birdshot chorioretinopathy (*n* = 4), toxoplasmosis (*n* = 1), herpes-related uveitis (*n* = 1) and pars planitis (*n* = 21)]. Overall, 126 patients met the inclusion criteria and were included for analyses. Baseline characteristics of the study population are summarized in Table 1. The majority were women (*n* = 77; 61%), with a median age of 49 [33–62] years. Most patients were of Caucasian origin (*n* = 113; 90%). The median follow-up duration was 20 months [3–59.5]. Anatomically, 87 patients (69%) had bilateral IU, which was almost always chronic (*n* = 125/126). Anterior segment involvement was observed in 10 cases (8%). Snowballs were observed in 39 cases (31%), snowbanks in 10 cases (8%), occurring together with snowballs in three out of four snowbank cases. Macular edema was found in 30 cases (24%), papillitis in 26 (21%), and venous vasculitis in 41 (33%). No cases of arterial vasculitis were identified. Table 2 shows these variables stratified by age group (< 30, 30–60, ≥ 60 years).

**Table 1:** General characteristics, ophthalmological findings, and etiological distribution in 126 patients

**Gender**		
Female	77	(61.1)
Male	49	(38.9)
**Ethnicity**		
Caucasian	113	(89.7)
North African	8	(6.3)
French overseas Caribbean	2	(1.6)
Sub-Saharan African	1	(0.8)
Middle Eastern	1	(0.8)
Mauritian origin	1	(0.8)
**Age**		
Years*	49.5	[33; 62]
**Follow-up duration**		
Months*	20	[3; 59.5]
**Characteristic of uveitis**		
Bilateral	87	(69.0)
Chronic	125	(99.2)
**Associated clinical signs**		
Anterior uveitis	10	(7.9)
Snowballs	39	(31.0)
Snowbanking	10	(7.9)
Macular edema	30	(23,8)
Papillitis	26	(20.6)
Venous vasculitis	41	(32.5)
**Etiology**		
*Idiopathic/Pars planitis*		
Idiopathic	66	(52.4)
Pars planitis	10	(7.9)
*Systemic*		
Sarcoidosis	20	(15.9)
Multiple Sclerosis	5	(4.0)
Radiologically isolated syndrome	1	(0.8)
Inflammatory Bowel disease	1	(0.8)
Behçet’s disease	1	(0.8)
*Infectious*		
Tuberculosis	2	(1.6)
Lyme	2	(1.6)
*Ophthalmological entities*		
Fuchs’ heterochromic cyclitis	3	(2.4)
Birdshot chorioretinopathy	4	(3.2)
*Masquerade* (Lymphoma)	11	(8.7)

Data are expressed as number and percentage, n (%), or * median [1st; 3rd quartile]

**Table 2:** General characteristics, ophthalmological findings, and etiological distribution of IU stratified by age group (< 30 years, 30–60 years, ≥ 60 years)

Characteristic	< 30 years (*n* = 20)	30–60 years (*n* = 71)	≥ 60 years (*n* = 35)
**Total cases**	20 (100%)	71 (100%)	35 (100%)
**Sex**			
Female	15 (75%)	37 (52%)	25 (71%)
Male	5 (25%)	34 (48%)	10 (29%)
**Ethnicity**			
Caucasian	19 (95%)	61 (86%)	33 (94%)
North African	1 (5%)	6 (8%)	1 (3%)
French overseas Caribbean	0 (0%)	1 (1%)	1 (3%)
Sub-Saharan African	0 (0%)	1 (1%)	0 (0%)
Middle-Eastern	0 (0%)	1 (1%)	0 (0%)
Mauritian origin	0 (0%)	1 (1%)	0 (0%)
**Laterality**			
Bilateral	16 (80%)	47 (66%)	24 (69%)
Unilateral	4 (20%)	24 (34%)	11 (31%)
**Course**			
Chronic	20 (100%)	71 (100%)	34 (97%)
Acute	0 (0%)	0 (0%)	1 (3%)
**Associated clinical signs**			
Anterior uveitis	3 (15%)	6 (8%)	1 (3%)
Snowballs	14 (70%)	22 (31%)	3 (9%)
Snowbanking	4 (20%)	6 (8%)	0 (0%)
Macular edema	6 (30%)	14 (20%)	10 (29%)
Papillitis	6 (30%)	14 (20%)	6 (17%)
Venous vasculitis	10 (50%)	25 (35%)	6 (17%)
**Etiology**			
Idiopathic	**10 (50%)**	**34 (48%)**	**22 (63%)**
Pars planitis	**7 (35%)**	3 (4%)	0 (0%)
*Systemic disease*			
Sarcoidosis	0 (0%)	**15 (21%)**	5 (14%)
Multiple sclerosis	2 (10%)	3 (4%)	0 (0%)
Radiologically isolated syndrome	1 (5%)	0 (0%)	0 (0%)
Inflammatory bowel disease	0 (0%)	1 (1%)	0 (0%)
Behçet’s disease	0 (0%)	1 (1%)	0 (0%)
*Infectious*			
Tuberculosis	0 (0%)	1 (1%)	1 (3%)
Lyme disease	0 (0%)	1 (1%)	1 (3%)
*Ophthalmic entities*			
Fuchs’ heterochromic cyclitis	0 (0%)	3 (4%)	0 (0%)
Birdshot chorioretinopathy	0 (0%)	4 (6%)	0 (0%)
*Other*			
Lymphoma	0 (0%)	5 (7%)	**6 (17%)**

Data are expressed as n (% of age group). The two most frequent etiologies per age group are shown in bold

### Etiological diagnosis

**Fig. 2:**
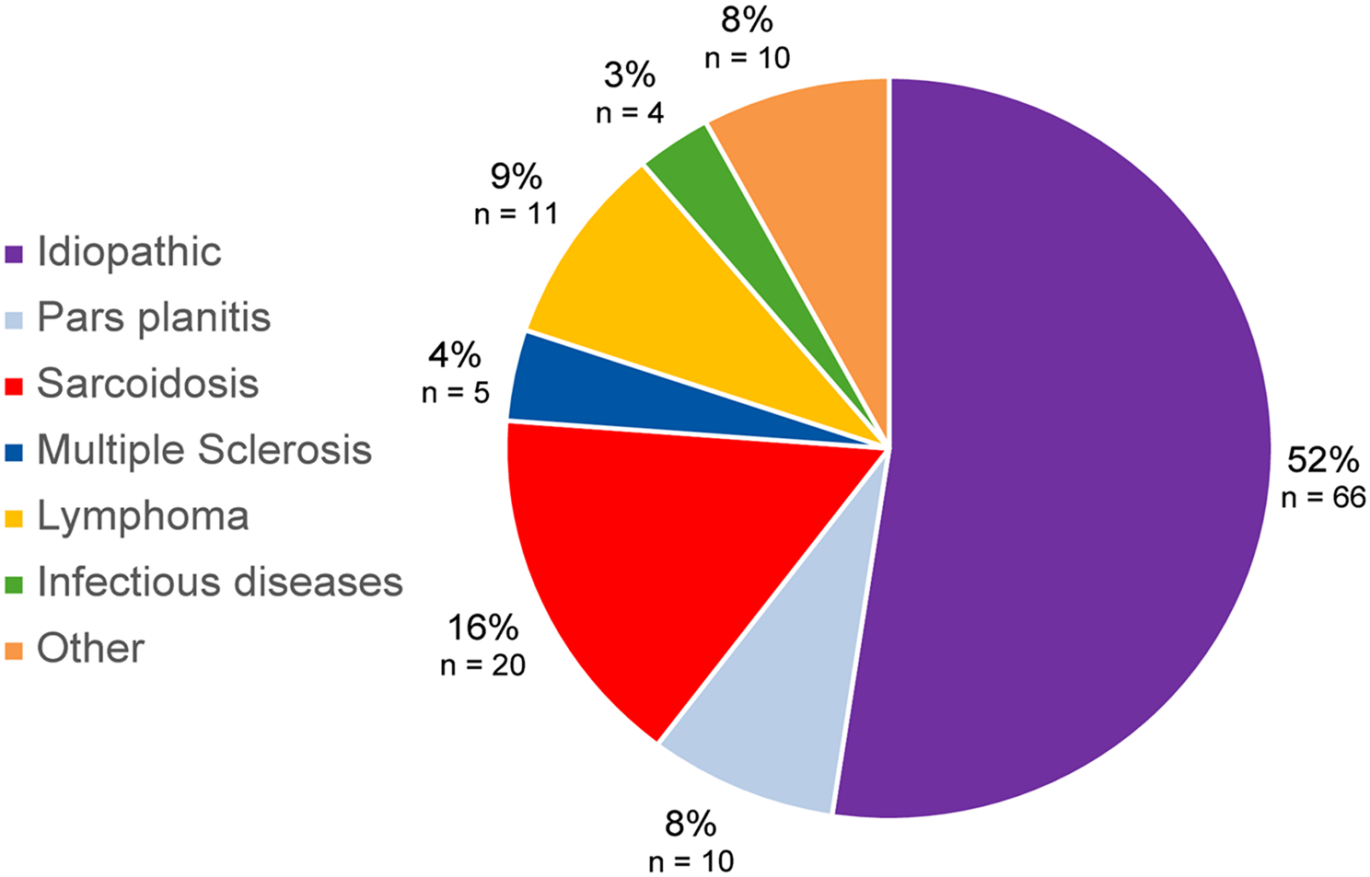
Etiology of IU

Uveitis was considered idiopathic in 66 patients (52.4%), with a median age of 50 [33–65] years. In non-idiopathic IU, the most frequently identified etiology was sarcoidosis (*n* = 20; 15.9%, median age: 54 [42–59] years), followed by lymphoma-associated IU (*n* = 11; 8.7%, median age: 62 [56.5–69.5] years), and pars planitis (*n* = 10; 7.8%, median age: 25 [22–30] years). Five patients (4%) had IU associated with multiple sclerosis (median age: 46 [26–49] years). Other etiologies included infectious IU (*n* = 4; 3.2% 2 related to tuberculosis and 2 to Lyme disease), Birdshot chorioretinopathy (*n* = 4; 3.2%), Fuchs’ heterochromic cyclitis (*n* = 3; 2.4%), Behçet’s disease (*n* = 1; 0.8%), inflammatory bowel disease (*n* = 1; 0.8%), and radiologically isolated syndrome (*n* = 1; 0.8%) (Fig. 2). Among the 11 cases of lymphoma-associated uveitis, all presented with predominant vitritis. Eight had isolated vitritis, two had vitritis associated with peripheral white retinal infiltrates, and one had vitritis associated with peripheral white infiltrates, papillitis, and venous vasculitis on FA, none presented sub retinal pigment epithelium deposit visible on macular cube OCT. Among the 13 non-Caucasian patients, distinct etiological profiles were observed. Eight originated from North Africa: three were diagnosed with sarcoidosis, one with Behçet’s disease, and four with idiopathic IU or pars planitis. One patient was from sub-Saharan Africa and was diagnosed with idiopathic IU. Two patients from French overseas territories had heterogeneous etiologies, namely idiopathic IU and inflammatory bowel disease–associated uveitis.

**Fig. 3:**
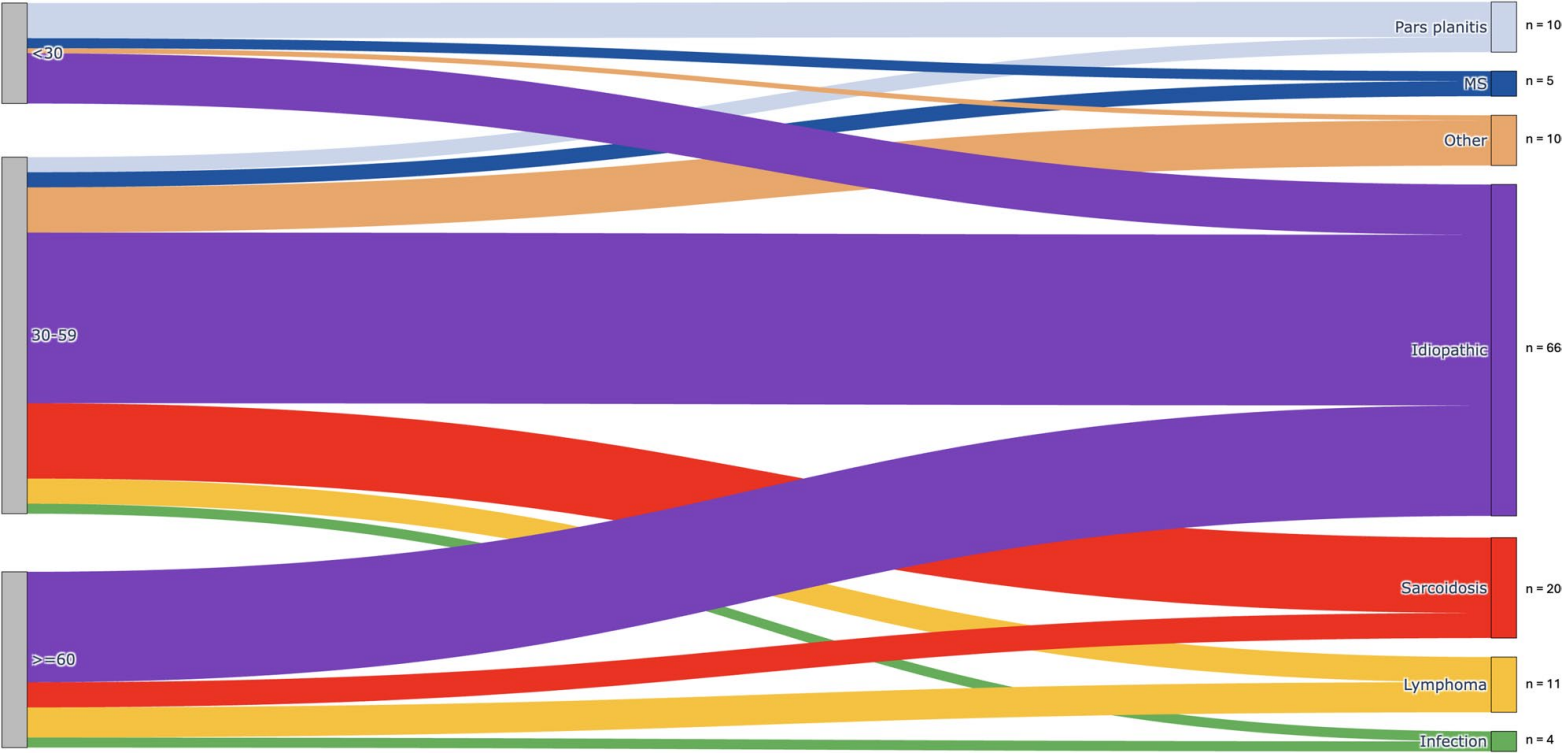
Distribution of the main intermediate uveitis etiologies across age groups

Etiologic distribution varied by age. In patients under 30, the most frequent etiologies were idiopathic uveitis, pars planitis, and multiple sclerosis. Between 30 and 60, idiopathic uveitis remained predominant, followed by sarcoidosis and lymphoma, the latter occurring only after age 45. After 60, idiopathic IU remained the leading cause, but lymphoma was more frequent than sarcoidosis. Figure 3 shows the distribution of the main intermediate uveitis etiologies across age groups.

The distribution of etiologies differed significantly across age groups (χ² = 35.8, df = 8, *p* < 0.001). The frequency of lymphoma demonstrated an age-related increase, whereas the prevalence of pars planitis declined with advancing age; however, neither trend reached statistical significance (trend analysis: *p* = 0.07 and *p* = 0.27, respectively). Sarcoidosis was more frequent in middle-aged patients (21%) compared with younger and older groups combined (9%), but the difference was not significant (*p* = 0.08).

### Diagnostic utility of complementary investigations

Regarding additional investigations: chest CT was performed in 122 patients (97%), and chest X-ray in 52 (41%). Both modalities were available for a total of 48 patients: 5 aged < 30, 30 aged 30–59, and 13 aged ≥ 60. Chest CT was contributive in 21 cases (17.2%), versus 7 for chest X-ray (13.5%). Among the 5 patients under 30, none had a CT scan positive when X-ray was negative, as no sarcoidosis cases were identified in this age group. Conversely, 4 patients aged 30–59 and 2 aged ≥ 60 had a positive CT despite a normal chest X-ray.

18 F-FDG PET/CT was performed in 47 patients (37%) and was positive in 13 cases (27.6%). In only one patient, it directly contributed to the diagnosis despite normal chest X-ray and CT findings: a 42-year-old man with chronic bilateral IU, presenting with posterior synechiae but no anterior granulomatous signs; the scan, prompted by corticosteroid dependence, revealed hyper-metabolic mediastinal lymph nodes.

Brain MRI was performed in 112 of 126 patients (89%) and was contributive in 10 cases (8.9%). Diagnoses included multiple sclerosis (*n* = 5), lymphoma (*n* = 4), and radiologically isolated syndrome (*n* = 1). One additional case was diagnosed with hydrocephalus, but with a prior diagnosis of sarcoidosis with positive chest CT. Two of the contributive MRIs (20%) were performed in asymptomatic patients: one revealing radiologically isolated syndrome, and the other oculocerebral lymphoma in a 50-year-old patient. According to age group, contributive MRIs led to three diagnoses in patients under 30 (one radiologically isolated syndrome and two multiple sclerosis), six in patients aged 30 to 59 (three multiple sclerosis and three lymphomas), and one lymphoma in a patient over 60.

Anterior chamber paracentesis with IL-6 and IL-10 analysis was performed in 62 of the 126 patients (49%). None of the patients had received systemic or local corticosteroid therapy before ACP. Ten cases of intraocular lymphoma (16%) were identified, each presenting an increase of IL10 level in the aqueous humor and a IL-6/IL-10 ratio > 1. No infectious uveitis case showed IL-10 elevation or a positive IL-10/IL-6 ratio. No positive results were found before age 30, since the youngest lymphoma case was 48 years old. In the 30–59 group, five positive ACPs involved patients aged 48 or above, while the remaining five occurred in patients over 60. One additional case of lymphoma had a negative ACP result, and the diagnosis was ultimately confirmed through vitrectomy. The patient was a 77-year-old with chronic intermediate uveitis manifesting as isolated bilateral vitritis; vitrectomy was undertaken because the intraocular inflammation remained unexplained in this elderly patient. No prior corticosteroid therapy had been administered that could have turned the ACP result negative. The diagnostic yield of each complementary investigation, including the specific contributive findings, is summarized in Table 3.

**Table 3:** Diagnostic utility of complementary investigations in 126 patients

Investigation	*n* performed (%)	Contributive results *n* (%)	Contributive findings
Chest X-ray	52 (41.3)	7 (15.5)	Hilar or mediastinal lymphadenopathies or pulmonary infiltration
Chest CT-scan	122 (96.8)	21 (17.2)	Hilar or mediastinal lymphadenopathies, perilymphatic nodules, or parenchymal abnormalities consistent with sarcoidosis
18F-FDG PET/CT	47 (37.3)	13 (27.6)	Focal hypermetabolic uptake compatible consistent with sarcoidosis
Brain MRI	112 (88.9)	10 (8.9)	Demyelinating lesions consistent with MS Lesions suggestive of lymphoma
Anterior chamber paracentesis	62 (49.2)	10 (16.1)	IL-10/IL-6 ratio > 1

Data are expressed as numbers and percentages, n (%)

## Discussion

With its large sample size, this real-life study provides a comprehensive overview of IU etiologies and their distribution across age groups, offering practical guidance for diagnostic strategies.

The demographic profile of our cohort is in line with major European studies, showing a predominance of female patients [4–6, 19]. Although no consistent gender pattern is established in IU, previous reports suggest that males are more frequently affected in childhood, whereas women are more often diagnosed in early adulthood and later in life [2]. Most patients were of Caucasian origin, which may have influenced the distribution of etiologies. As described in the literature, certain causes of uveitis vary by ethnicity and geography: Behçet’s disease represents a leading cause of uveitis in Mediterranean populations; however, it rarely presents as isolated IU [20], while IU related to tuberculosis is encountered predominantly in patients from endemic regions [21–23]. Although non-Caucasian patients were few, their etiologic profiles differed from those of Caucasian individuals. This highlights the need to consider ethnic and geographical background when evaluating IU. In particular, sarcoidosis or tuberculosis should be more strongly considered in patients from North Africa or sub-Saharan regions, whereas Behçet’s disease is more often associated with Mediterranean ancestry [11]. Such epidemiological factors may help refine diagnostic priorities in non-Caucasian patients.

Only a limited number of major European cohorts have specifically reported the age distribution of intermediate uveitis. Among those that did, our study stands out with a higher median age (49 years), compared to a median age of 26 years in the Italian cohort by Luca et al. [19], a mean age of 35 years in the German series by Ness et al. [6], and a median age of 44 years in the German cohort by Grajewski et al. [5]. This discrepancy may be explained by several factors. First, our cohort included cases of lymphoma-associated IU, a condition typically affecting older individuals and was excluded in the Italian cohort (Luca et al. [19]). Second, the inclusion of pediatric cases in those cohorts likely contributed to lowering their reported mean or median age [5, 6, 19].

When considering all age groups, the overall etiological distribution in our cohort is broadly similar to that reported in previous European studies, with idiopathic cases being the most frequent, followed by systemic causes, mainly sarcoidosis and MS [3–6, 24, 25]. As in other Western European cohorts, IU related to tuberculosis was rare, reflecting the low prevalence of this infection in non-endemic regions [6]. We acknowledge that, according to the SUN classification, Fuchs’ heterochromic cyclitis is categorized as anterior uveitis [26] and Birdshot chorioretinopathy as posterior uveitis [27]. However, within the real-life context of our study, these cases were referred to internal medicine as unexplained intermediate uveitis due to predominant vitreous involvement. Their inclusion therefore reflects the pragmatic referral patterns and diagnostic pathways encountered in tertiary care rather than a strict taxonomic reclassification. These ophthalmologic entities were also underrepresented, as we excluded intermediate uveitis cases whose etiology had been established solely through ophthalmologic assessment. In contrast, intraocular lymphoma was overrepresented in our series. As previously discussed, this likely reflects the real-life nature of our study, which included all patients referred with a clinical presentation of IU, regardless of the final diagnosis. Since lymphoma is often excluded or classified as a masquerade syndrome in other studies [3, 19, 24, 25], its inclusion here reflects our intent to capture the full clinical spectrum encountered in practice.

We divided patients with IU into three age groups: under 30 years, 30 to 60 years, and over 60 years. This stratification reflects age-related immunological changes and well-documented shifts in etiological patterns across the lifespan [6, 28–32]: in patients under 30, IU is most often idiopathic, typically presenting as pars planitis, which is rarely reported beyond this age [6, 28]. From the age of 30, systemic immune-mediated conditions, particularly sarcoidosis, become more frequent [6]. Patients over 60 form a distinct subgroup: IU is still often idiopathic, but sarcoidosis, infections, and masquerade syndromes (e.g., intraocular lymphoma) are more common, possibly linked to age-related declines in cell-mediated immunity [29–32]. This stratification facilitates a more targeted etiological work-up and improves comparability with existing data. However, we considered 45 years as a clinically meaningful threshold to orient lymphoma-directed investigations. This choice is supported by epidemiological data showing that primary vitreoretinal lymphoma is exceptionally rare before the age of 50, with mean or median ages at diagnosis around 60–63 years in large international series [33–35]. Using a 45-year threshold therefore allows earlier identification of the rare younger cases reported in the literature, while remaining consistent with established epidemiological patterns. In our cohort, idiopathic uveitis, pars planitis, and MS were predominant in patients under 30. Between 30 and 60 years, idiopathic forms remained common, but sarcoidosis and lymphoma emerged as leading causes. After 60, idiopathic uveitis remained the most frequent, while lymphoma became more common than sarcoidosis.

Pars planitis was mostly observed in patients under 30 (27%) and was rare in older groups. This distribution is in line with its typical onset in younger individuals and its known contribution to pediatric uveitis [10].

Sarcoidosis was the most common systemic etiology, affecting mainly patients aged 40–60 years in our cohort, with no case before age 30. Multiple sclerosis ranked second, with most diagnoses in the second to third decades; a few cases occurred later, but none beyond age 60. This age pattern mirrors other epidemiological series: in the German tertiary-center cohort of Ness et al. [6], the mean age at diagnosis was 44 years for sarcoidosis-associated IU and 37 years for MS-associated IU; in the multinational SUN Working-Group dataset, the median age for sarcoidosis-related IU was 52 years (IQR 43–67) [36], whereas MS-associated IU showed a median age of 37 years (IQR 30–48) [37]. These external data therefore corroborate that sarcoidosis tends to present later than multiple sclerosis in intermediate uveitis.

Intraocular lymphoma should be considered in patients over 40 with chronic bilateral IU, particularly when inflammation recurs or shows partial or transient corticosteroid responsiveness [7, 38]. In our study, no cases were identified before the age of 45. This observation is corroborated by the literature: a German multicentre series of 22 patients with vitreoretinal lymphoma —manifesting predominantly as hyalitis— reported a median age at diagnosis of 64 years (range 38–83 years), with every patient aged ≥ 45 years except for a single 38-year-old case [39].

As in reported in the literature [6], infectious causes predominantly affected older patients in our study.

Among the 48 patients who underwent both chest X-ray and CT, no discordance was observed in those under 30, which is consistent with the absence of sarcoidosis in this age group. In contrast, 4 discordant cases were identified between 30 and 59 years and 2 in patients aged 60 or above. These findings align with Borciuch et al. [14], who reported low discordance rates in younger patients, especially with normal ACE, and a marked increase with age, supporting a targeted use of CT in older individuals.

The diagnostic yield of 18 F-FDG PET/CT was limited in our cohort: although 13 scans demonstrated hyper-metabolic foci, only one led to an etiological diagnosis that was not suspected based on conventional imaging. No PET/CT was contributive in patients under 30, as no sarcoidosis was identified in this age group. Rahmi et al. [40] reported that approximately one-third of patients with chronic uveitis had a positive PET/CT, with more than half of these cases showing abnormalities despite a normal chest CT. PET/CT positivity was associated with older age, posterior synechiae, and abnormal chest imaging, highlighting its usefulness in detecting subclinical or extrapulmonary sarcoidosis. Similarly, Chauvelot et al. [41], focusing on patients with normal chest CT, identified older age at uveitis onset, posterior synechiae, and elevated ACE levels as independent predictors of PET/CT positivity. These findings, consistent with our data, support the highly selective use of PET/CT as a second-line diagnostic tool in older patients with compatible clinical signs and inconclusive first-line imaging, especially considering that PET/CT was not performed in all patients and contributed to a definitive diagnosis in only one case.

In our cohort of intermediate uveitis, brain MRI was contributive in 8.9% of cases, but only one etiological diagnosis—oculocerebral lymphoma—was made in an asymptomatic patient No cases of multiple sclerosis were diagnosed based on brain MRI in patients without neurological symptoms. These findings support a selective use of brain MRI, particularly in patients presenting with neurological signs or clinical features suggestive of oculocerebral lymphoma, such as age over 40 [42].

Anterior chamber paracentesis also proved useful in several positive cases. However, one patient with a negative result was ultimately diagnosed with lymphoma. This highlights the possibility of false negatives, a limitation also reported by Pochat-Cotilloux et al. [43], who described early-stage lymphomas with undetectable IL-10 levels, potentially due to low tumor burden or limited cytokine secretion. These findings underscore the importance of repeated testing or proceeding to diagnostic vitrectomy when clinical suspicion remains high.

**Fig. 4:**
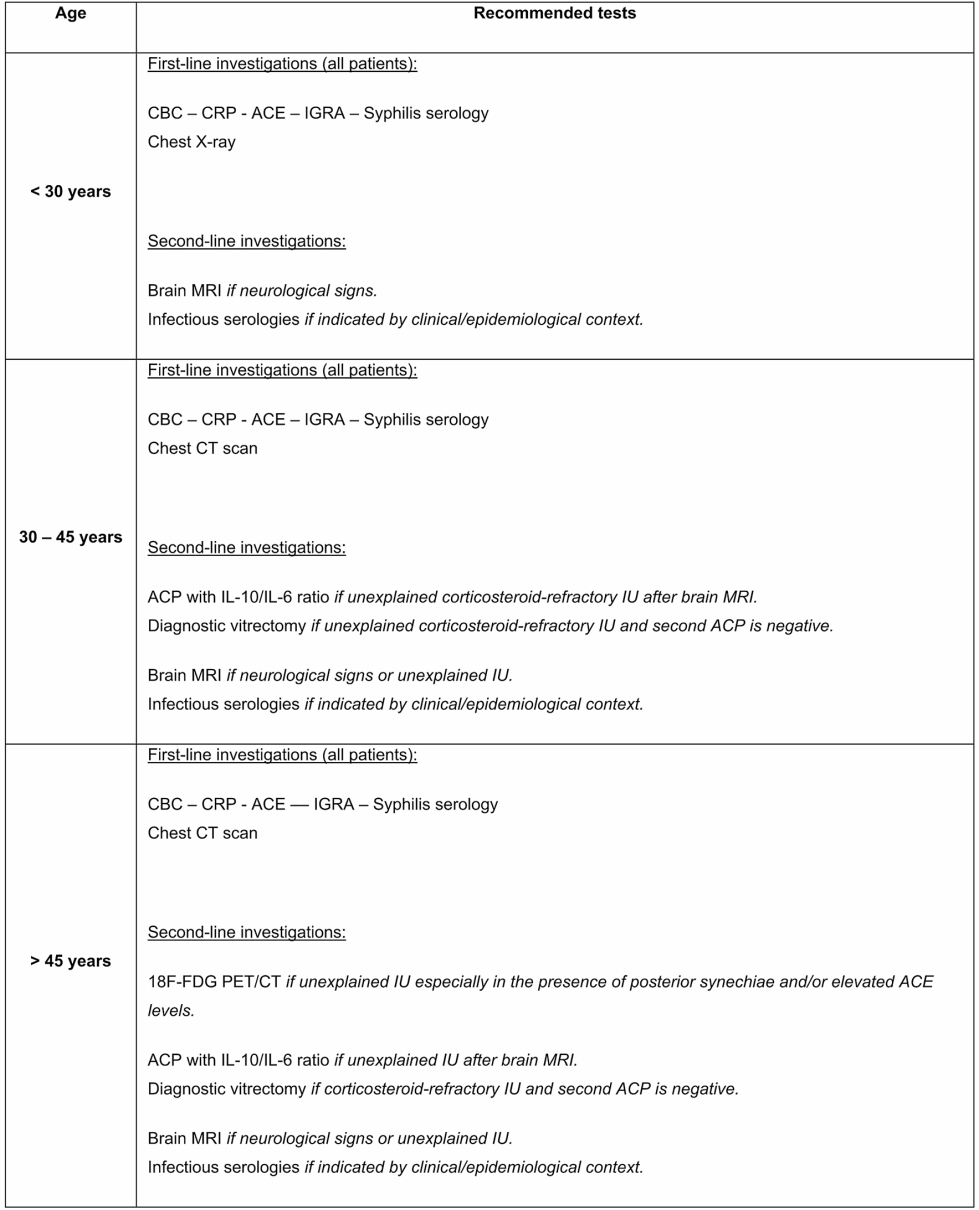
Age-based diagnostic work-up for intermediate uveitis. ACE = angiotensin-converting enzyme; ACP = anterior chamber paracentesis; CBC = complete blood count; CRP = C-reactive protein; IGRA = interferon-gamma release assay; IU = intermediate uveitis; 18 F-FDG PET/CT = positron emission tomography/computed tomography using fluorine-18 fluorodeoxyglucose. In this diagnostic algorithm, an investigation is considered contributive when its findings help guide the etiologic evaluation. Examples include: Chest imaging (X-ray or CT): bilateral hilar and/or mediastinal lymphadenopathy suggesting sarcoidosis. Brain MRI: demyelinating lesions compatible with multiple sclerosis, or imaging features suggestive of lymphoma. 18 F-FDG PET/CT: hypermetabolic lymph nodes or focal uptake indicating granulomatous or lymphoproliferative involvement. ACP with IL-10/IL-6 analysis: an IL-10/IL-6 ratio > 1 supporting the suspicion of intraocular lymphoma. Corticosteroid-refractory IU is defined as inflammation persisting despite > 1 mg/kg/day of systemic corticosteroids for ≥ 1 month

**Fig. 5:**
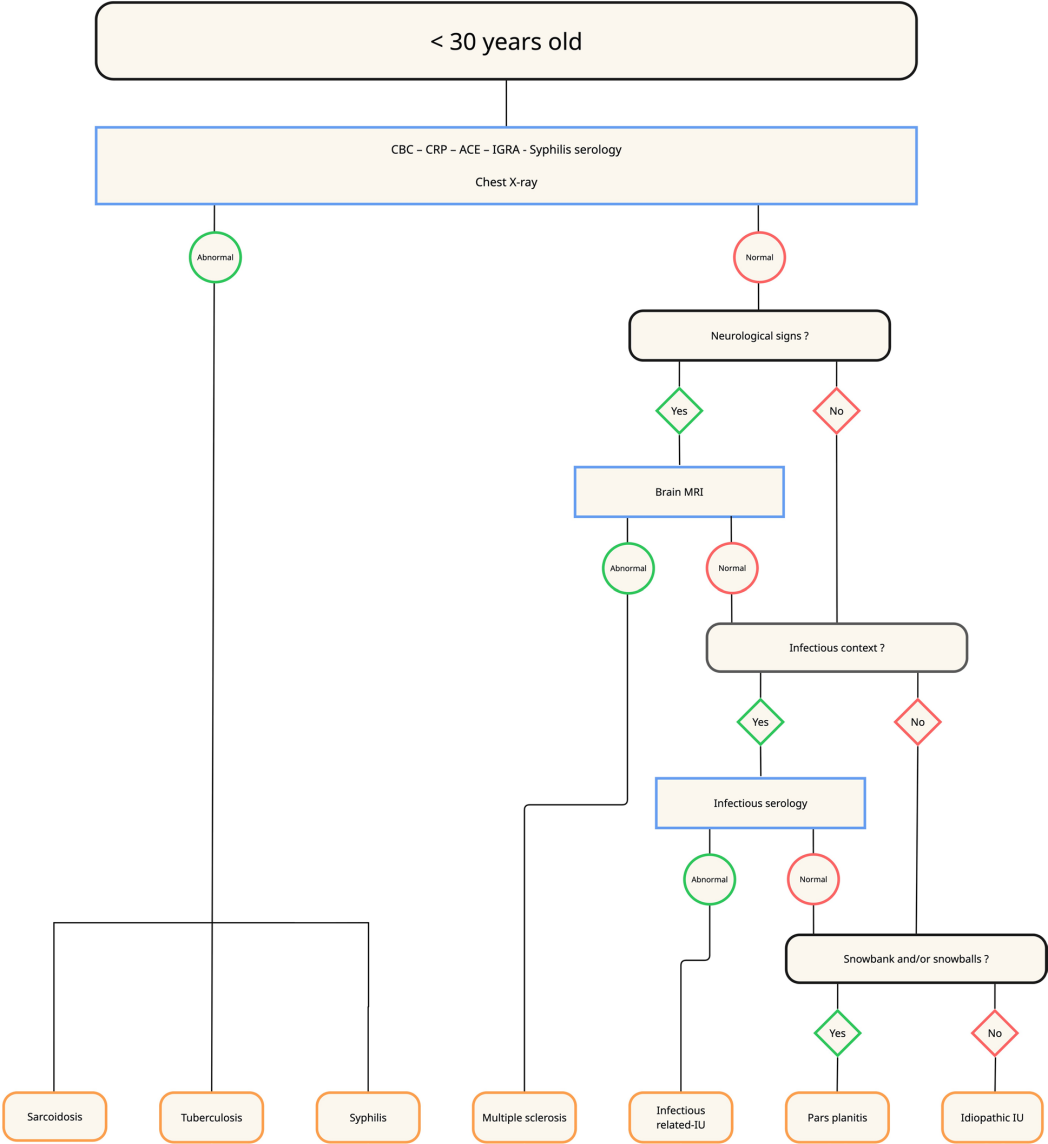
Diagnostic algorithm for intermediate uveitis in patients younger than 30 years

**Fig. 6:**
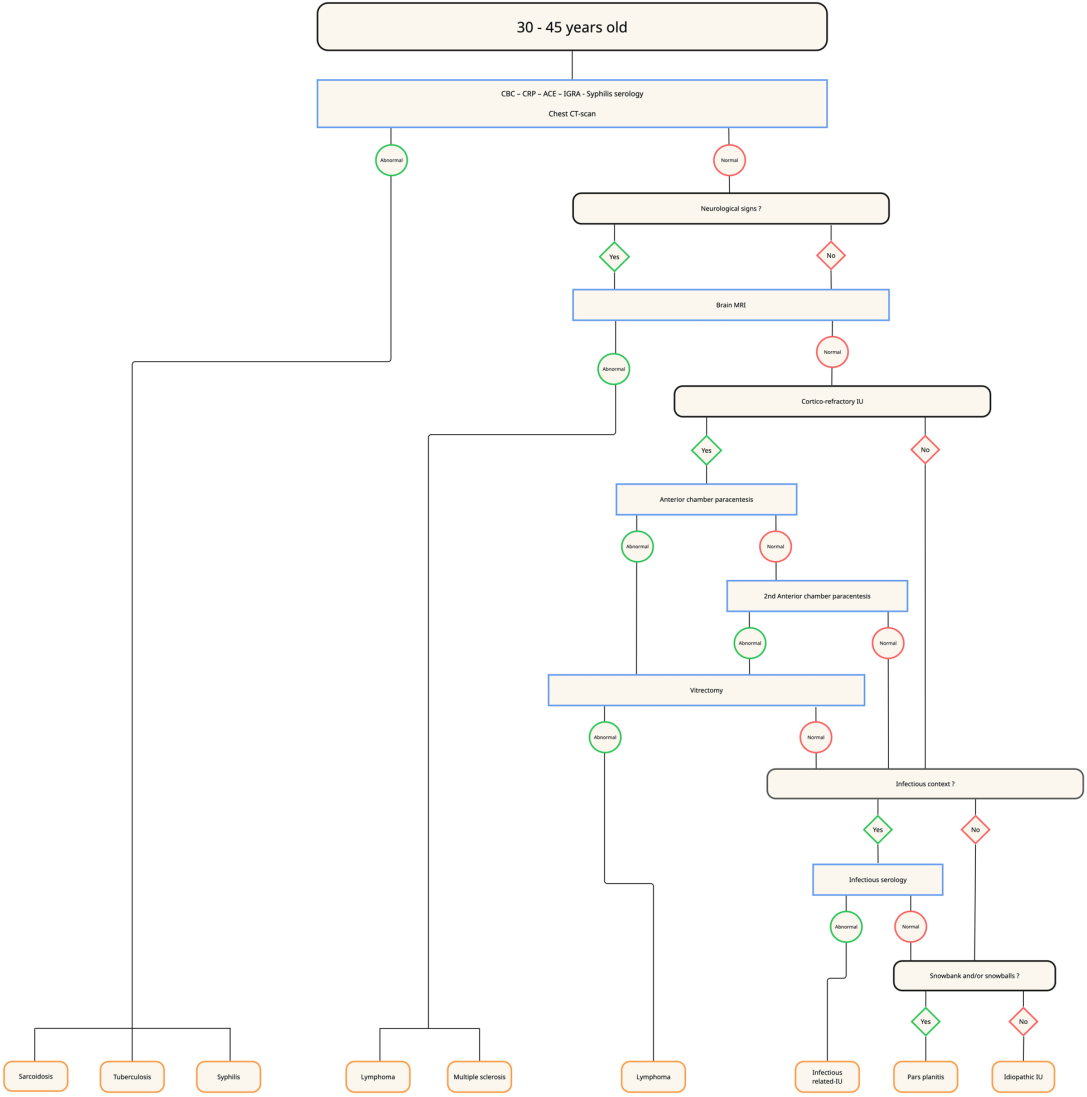
Diagnostic algorithm for intermediate uveitis in patients aged 30 to 45 years

**Fig. 7:**
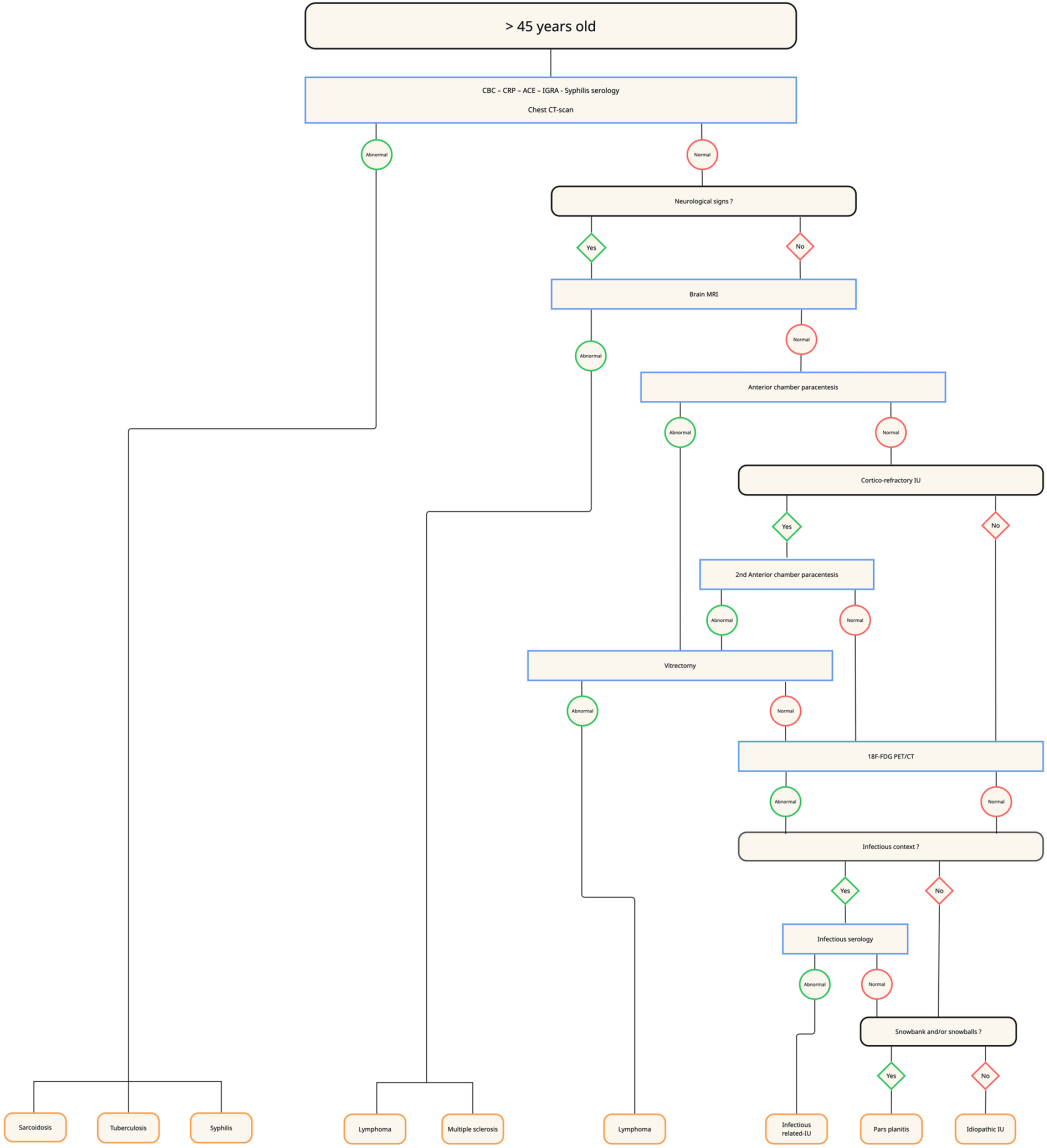
Diagnostic algorithm for intermediate uveitis in patients older than 45 years

The ULISSE study highlighted the relevance of a standardized diagnostic approach, particularly based on the anatomical type of uveitis [12], which supported expert recommendations for a structured diagnostic strategy [44]. Building on these findings, along with our own data and the current literature, we propose an age-adapted diagnostic work-up for intermediate uveitis (Figs. 4, 5, 6 and 7). We opted for a 45-year cut-off rather than 60 years, as our data showed a marked increase in lymphoma incidence beyond this age -warranting systematic anterior chamber paracentesis in all unexplained IU from 45 years onward.

- First-line laboratory evaluation (all age groups) mirror ULISSE — CBC, CRP, ACE, IGRA, and syphilis serology.
- Chest imaging — in patients younger than 30 years, a chest X-ray is sufficient; from 30 years onward, the radiograph is replaced by a chest CT scan.
- Brain MRI — before 30 years, performed only when neurological signs are present; between 30 and 45 years, also indicated in corticosteroid-refractory IU; after 45 years, used chiefly to rule out oculo-cerebral lymphoma. Corticosteroid-refractory IU is defined as inflammation persisting despite >1 mg/kg/day of systemic corticosteroids for ≥ 1 month [45].
- ¹⁸F-FDG PET/CT — considered as a second-line investigation when IU remains unexplained, particularly in patients ≥ 45 years with posterior synechiae and/or an elevated ACE level.
- Anterior-chamber paracentesis (IL-10/IL-6 analysis) — recommended in unexplained corticosteroid-refractory IU between 30 and 45 years and performed systematically in unexplained IU after 45 years when chest CT and brain MRI are normal; a negative but clinically suspicious result warrants repeat sampling or diagnostic vitrectomy.
- Additional infectious or immunological tests — ordered only when justified by specific clinical or epidemiological indicators.

This study presents several limitations. Its retrospective, monocentric design may limit generalizability. Conducted in France in a largely Caucasian cohort, this study reflects a regional epidemiology with fewer infectious causes, which may limit the applicability of our findings to more diverse or tuberculosis-endemic regions. Therefore, the proposed age-based diagnostic strategy might require regional adaptation, with earlier emphasis on infectious screening in endemic settings. Finally, the tertiary setting may have introduced selection bias, with certain etiologies, such as sarcoidosis and intraocular lymphoma, being overrepresented due to referral of unresolved or atypical cases. Nonetheless, the real-life context strengthens the clinical relevance of our findings and supports an age-based diagnostic approach in IU.

## Conclusion

Age is a key factor to guide the diagnostic strategy in intermediate uveitis. Based on our study and a literature analysis, we suggest an age-tailored diagnostic strategy that could improve efficiency by reducing unnecessary investigations in low-risk patients while streamlining the diagnostic pathway in high-risk cases. Its prospective validation in real-life conditions would be essential to confirm both its clinical relevance and practical impact.

## Data Availability

Data is provided within the manuscript.
